# The return of the lesion for localization and therapy

**DOI:** 10.1093/brain/awad123

**Published:** 2023-04-11

**Authors:** Juho Joutsa, Nir Lipsman, Andreas Horn, G Rees Cosgrove, Michael D Fox

**Affiliations:** Turku Brain and Mind Center, Clinical Neurosciences, University of Turku, 20014 Turku, Finland; Turku PET Centre, Neurocenter, Turku University Hospital, 20520 Turku, Finland; Center for Brain Circuit Therapeutics, Departments of Neurology, Psychiatry, and Radiology, Brigham and Women’s Hospital, Harvard Medical School, Boston, MA 02115, USA; Division of Neurosurgery, Sunnybrook Health Sciences Centre, University of Toronto, Toronto, ON M4N 3M5, Canada; Hurvitz Brain Sciences Research Program, Sunnybrook Research Institute, Sunnybrook Health Sciences Centre, University of Toronto, Toronto, ON M4N 3M5, Canada; Harquail Centre for Neuromodulation, Sunnybrook Research Institute, Toronto, ON M4N 3M5, Canada; Center for Brain Circuit Therapeutics, Departments of Neurology, Psychiatry, and Radiology, Brigham and Women’s Hospital, Harvard Medical School, Boston, MA 02115, USA; Movement Disorder and Neuromodulation Unit, Department of Neurology, Charité - Universitätsmedizin Berlin, 10117 Berlin, Germany; Department of Neurology, Massachusetts General Hospital, Harvard Medical School, Boston, MA 02114, USA; Department of Neurosurgery, Massachusetts General Hospital, Harvard Medical School, Boston, MA 02114, USA; Center for Brain Circuit Therapeutics, Departments of Neurology, Psychiatry, and Radiology, Brigham and Women’s Hospital, Harvard Medical School, Boston, MA 02115, USA; Department of Neurosurgery, Brigham and Women’s Hospital, Harvard Medical School, Boston, MA 02115, USA; Center for Brain Circuit Therapeutics, Departments of Neurology, Psychiatry, and Radiology, Brigham and Women’s Hospital, Harvard Medical School, Boston, MA 02115, USA; Department of Neurology, Massachusetts General Hospital, Harvard Medical School, Boston, MA 02114, USA; Department of Radiology, Massachusetts General Hospital, Harvard Medical School, Boston, MA 02114, USA

**Keywords:** stroke, lesion mapping, lesion network mapping, connectivity, MRgFUS

## Abstract

Historically, pathological brain lesions provided the foundation for localization of symptoms and therapeutic lesions were used as a treatment for brain diseases. New medications, functional neuroimaging and deep brain stimulation have led to a decline in lesions in the past few decades. However, recent advances have improved our ability to localize lesion-induced symptoms, including localization to brain circuits rather than individual brain regions. Improved localization can lead to more precise treatment targets, which may mitigate traditional advantages of deep brain stimulation over lesions such as reversibility and tunability. New tools for creating therapeutic brain lesions such as high intensity focused ultrasound allow for lesions to be placed without a skin incision and are already in clinical use for tremor. Although there are limitations, and caution is warranted, improvements in lesion-based localization are refining our therapeutic targets and improved technology is providing new ways to create therapeutic lesions, which together may facilitate the return of the lesion.

## Teaser

Historically, lesions were fundamental to localizing and treating neurological symptoms; however, their use decreased with the adoption of techniques such as functional imaging and electrical neuromodulation. Joutsa *et al*. argue that further improvements in technology mean that lesions may be making a comeback in the treatment of brain disease.

## Introduction

**Figure 1:**
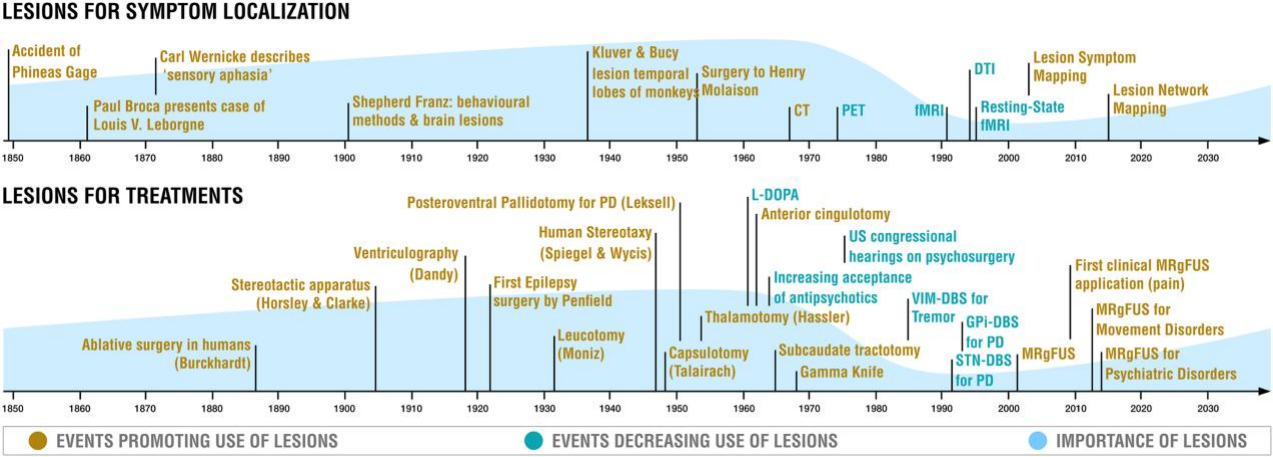
**Timeline of selected events illustrating the role of lesions in neuroscience and medicine**. *Top*: Spontaneously occurring lesions played a defining role in mapping human brain function, including localization of neurological and psychiatric symptoms. The role of lesions declined with the advent of functional imaging techniques such as PET and functional MRI (fMRI) but is now on the rise due to modern lesion mapping techniques. *Bottom*: Brain lesions have been used to treat neurological and psychiatric symptoms for nearly one and a half centuries. The therapeutic role of lesions decreased with the development of effective medications and deep brain stimulation (DBS) but is now on the rise due to modern lesioning technologies such as magnetic resonance-guided focused ultrasound (MRgFUS). Note that this timeline is not intended to be comprehensive, but to illustrate the rise, fall and return of lesions. There are exceptions to these historical trends, including DBS treatments explored in 1950s,^1,2^ subthalamic nucleus lesions in 1990,^3^ and many lesion studies that occurred during the epoch of functional neuroimaging.^4^ DTI = diffusion tensor imaging; PD = Parkinson's disease.

Historically, pathological lesions played a key role in localization and therapeutic lesions in treatment of neurological and psychiatric symptoms (Fig. 1). Over time, the number of lesion studies declined as newer technologies emerged, including functional neuroimaging (for localization) and electrical neuromodulation (for treatment). Despite important limitations, lesions have recently begun to make a comeback, aiding localization for a variety of different symptoms and as a clinical therapy in movement disorders. Here, we provide a personal perspective on why lesions are making a comeback and why they could return as an important treatment option for brain disease.

### History of brain lesions in neuroscience and medicine

Brain lesions have been used to localize and treat neurological and psychiatric symptoms for well over a century (Fig. 1). Early lesion-based localization was based on individual patients with pathological lesions such as Phineas Gage, Victor Leborgne (Tan) and Henry Gustav Molaison (patient H.M.). These case studies helped localize social inhibition to the prefrontal cortex, speech production to the inferior frontal lobe and memory to the hippocampi. Lesion-based studies increased in power and popularity with the advent of structural brain imaging in the 1970s, allowing for localization of lesion-induced deficits *in vivo*.^5,6^ However, by the 1980s, lesion-based studies were on the decline, in part due to functional neuroimaging technologies like PET and functional MRI (fMRI). These technologies allowed for localization of brain function in patients without brain lesions and helped overcome some of the challenges of lesion studies such as difficulty recruiting sufficient numbers of patients with similar symptoms or lesion locations, lack of statistical power and biological variability of spontaneous lesions in size and pathological process (e.g. lesion type, temporal evolution of the lesion and symptoms, perilesional effects and potential compensatory effects and plasticity). Owing to this added flexibility, functional neuroimaging studies soon became the dominant tool for localization of human brain function, greatly exceeding the number of lesion-based studies.^7,8^ Advances in invasive animal studies, including large consortia (such as the International Brain Laboratory) and new techniques (such as optogenetics), together with increasing use of computational models (such as deep neural networks) may have further contributed to the decline in lesion studies.

Therapeutic lesions were also leveraged for treatment across psychiatric and neurological conditions (Supplementary Table 1). In the 1920s–40s, surgical lesions were used to treat many conditions including epilepsy (temporal lobectomy),^9^ Parkinson’s disease (corticectomy, pedunculotomy, cordotomy) and psychiatric disorders (leucotomy or lobotomy).^10^ Many of these early procedures were limited in efficacy, associated with unacceptable side-effects, and led to ethical concerns, especially in the case of psychosurgery.^10,11^ Serendipitous findings, such as improvement in tremor following an accidental iatrogenic thalamic lesion,^12^ and adoption of the stereotactic frame led to more reproducible lesions with fewer side effects. Randomized controlled trials soon validated the efficacy of therapeutic lesions for movement disorders.^13^ However, by the 1980s, lesion-based treatments were on the decline, in part due to the development of better pharmacological treatments^10,14,15^ and the finding that similar therapeutic benefit could obtained by electrical stimulation of traditional lesion targets.^16^ The latter finding led to the development of deep brain stimulation (DBS), which had the advantage of reversibility and tunability with less side-effects compared to therapeutic lesions, especially for bilateral interventions.^16^ A head to head trial of DBS versus thalamotomy for tremor highlighted these advantages, and by 2010, the number of DBS procedures greatly exceeded the number of lesion procedures for movement disorders.^17^

## Lesion-based localization of symptoms

### Why consider returning to pathological lesions for localization?

Since the introduction of functional neuroimaging techniques such as PET^18^ and fMRI,^19^ these tools have dominated efforts to map symptoms to human neuroanatomy. The number of studies utilizing these technologies has dwarfed the number of lesion-based studies in the past few decades.

However, lesion studies have one important advantage over functional neuroimaging in that they can allow for causal links between the location of the lesion and the resulting symptoms.^20^ This weakness of functional neuroimaging has been referred to as the ‘causality gap’ and may limit the ability to translate neuroimaging findings into therapeutic targets.^21-23^ A return to lesion-based localization may help address this weakness, allowing for stronger causal inference in symptom localization.^20^ For example, finding that a lesion of structure X impairs process Y shows that lesioning X is sufficient to cause impairment of Y. However, it should be noted that this does not mean that lesion of X is necessary for causing deficits of Y.^20^

Lesions are also valuable to critically test and validate neuroimaging findings, and vice versa.^24,25^ If the results do not align between these two complementary approaches, we need to rethink the data and its interpretation. However, these two approaches provide complementary information as lesion studies can identify brain regions essential to function whereas neuroimaging studies identify regions that are involved in, but not necessarily essential to, a particular brain function.

### Recent advances in lesion mapping

The causal inference allowed by lesion studies is bolstered by recent advances in lesion mapping. While early lesion-based localization was based on individual patients, modern lesion studies often include hundreds of patients, enabled by the interconnected age of information technology, and the use of advanced statistics to better map lesion-induced deficits to neuroanatomy.^26,27^ For example, a recent study of nearly 500 stroke patients showed that language deficits could be linked to damage of specific locations in the right hemisphere, not just the left hemisphere.^28^ As methods and sample sizes have improved, it has become clear that lesion-induced deficits often fail to map onto single brain regions. For example, amnesia can be caused by lesions outside the hippocampus, hemichorea by lesions outside the subthalamic nucleus, and hemiparkinsonism by lesions outside the nigrostriatal tract.^20,29^ In these cases, lesion-based localization can benefit from incorporating information about brain connectivity.^29^

Atlases of human brain connectivity are now available, built from thousands of subjects scanned with techniques such as resting state functional connectivity MRI and diffusion tensor imaging.^30-32^ Using these circuit maps, one can map lesion-induced effects to specific white matter connections or functionally connected brain networks, rather than individual brain regions. Referred to as lesion network mapping^29^ or disconnection mapping,^33,34^ this approach has proven valuable across a wide range of neurological and psychiatric symptoms. For example, lesions causing amnesia, hemichorea and parkinsonism all fail to map to single brain regions but do map to specific brain networks.^20,29^

### Translating advances in lesion-based localization into treatment

**Figure 2:**
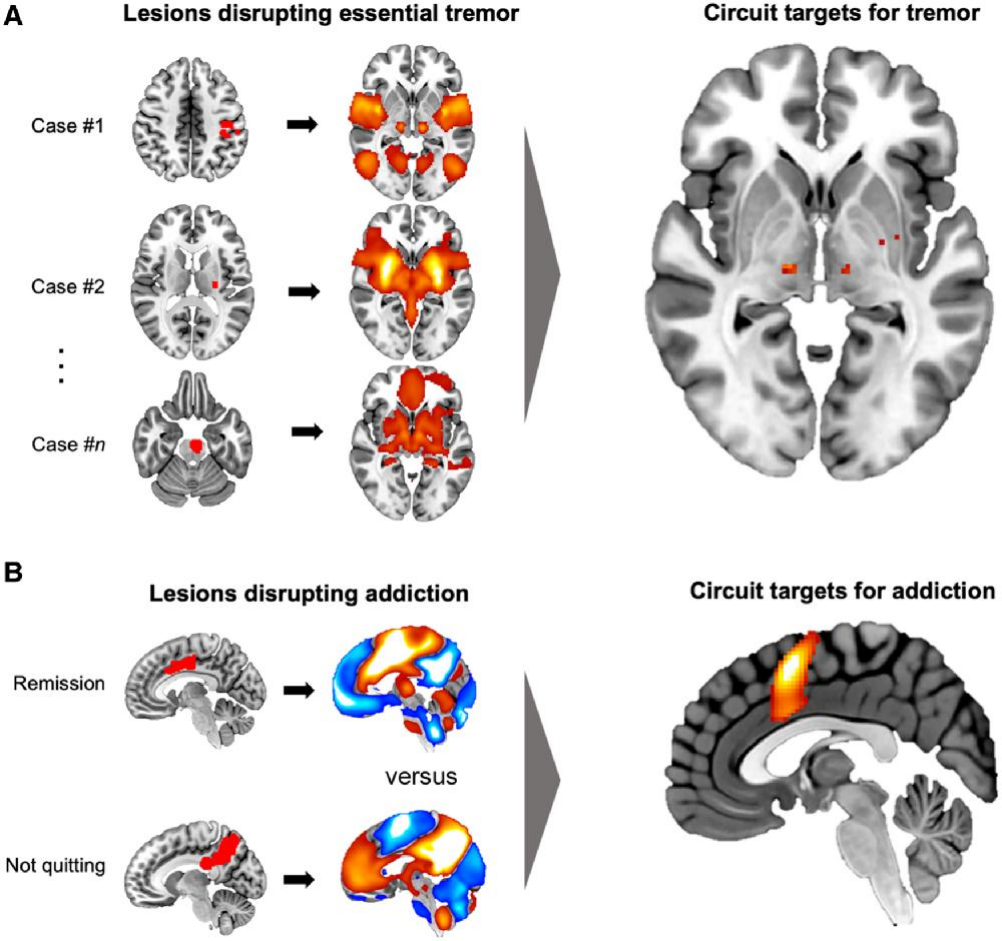
**Using brain circuit data to improve lesion-based localization and treatment**. (**A**) Lesions resulting in essential tremor relief (*left*). Using normative connectome data, functional connectivity between each lesion location and the rest of the brain can be computed (*middle*). Brain regions connected to all lesion locations disrupting tremor can then be identified, identifying peaks in the ventral intermediate nucleus (VIM) of the thalamus (the current main therapeutic target for tremor; *right*). Modified with permission from Joutsa *et al*.^35^ (**B**) Examples of lesions that did or did not result in remission of smoking addiction (*left*) with their corresponding connectivity profiles (*middle*). Brain voxels best representing the connectivity difference between lesion locations disrupting addiction versus lesion locations in patients who continued smoking included the insula/opercular region and paracingulate cortex (*right*). Figure modified from Joutsa *et al*.^42^ Image distributed under a Attribution 4.0 International (CC BY 4.0) license.

Advances in lesion-based symptom localization may translate into better therapeutic targets for neuromodulation or lesion-based treatments. Perhaps the most straight-forward example for such translation is localizing symptom improvement after spontaneous brain lesions.^35^ Although rare, spontaneous brain lesions have been reported to improve drug addiction,^36^ movement disorders,^35,37^ stuttering,^38^ pain,^39^ tinnitus^40^ and even foreign accent syndrome.^41^ These spontaneous but beneficial brain lesions might help identify lesion-based treatment targets.^35^ For example, a proof-of-concept study examined eleven lesion locations resulting in tremor improvement. These lesions occurred in different brain locations, but they were all part of a single connected brain circuit with a hub in the ventral intermediate nucleus of thalamus. This hub aligned perfectly with the primary lesion and DBS target for treating tremor (Fig. 2A).^35^ A recent study used this same approach to identify a brain circuit mediating smoking addiction remission (Fig. 2B).^42^ This circuit generalized to other substances of abuse and aligned with prior therapeutic targets from both surgical lesions and non-invasive brain stimulation. Future work will determine if the refined therapeutic targets identified in this study lead to improved efficacy for treatment of addiction.

Improved localization of lesion-based symptoms may also help to avoid side effects of lesion-based treatments. For example, lesions that cause depression map to a specific brain circuit with a hub in the left dorsolateral prefrontal cortex and lesions that cause memory impairment map to the circuit of Papez, including the hippocampus.^43,44^ DBS sites that cause depression or cognitive impairment in patients with Parkinson’s disease are connected to these same circuits.^45,46^ As such, avoiding these circuits could help guide DBS programming, but could be even more important in lesion-based treatments where side effects may be irreversible.

Finally, lesions that cause a symptom could help identify or refine therapeutic targets for relief of that symptom. Lesion locations causing parkinsonism, dystonia and Holmes tremor each map to distinct brain circuits. In each case, DBS sites that improve these symptoms are connected to the same brain circuit as lesions that caused these symptoms.^47-49^

There remain important challenges for translating advances in lesion-based localization into therapeutic targets. First, there is no guarantee that lesions ‘causing’ a symptom will identify the best neuroanatomical target for ‘improving’ that symptom.^29^ For example, brain functions lost due to brain damage may not be reversible and treatment should instead target brain circuits that could help compensate for the loss of function. Second, if seeking to map lesion locations to brain circuits, it remains unknown if one should use an atlas of anatomical connectivity, functional connectivity, or some combination of the two.^33,50,51^ Finally, if lesions localize to a brain circuit, it is unclear whether one should target a specific node in that circuit or a tract connecting multiple nodes of the circuit, such as the cerebellothalamic pathway in essential tremor.^52,53^

## Lesion-based treatment

### Why consider returning to lesions for treatment?

Although therapeutic lesions have been used as a treatment for a variety of neurological and psychiatric conditions (Supplementary Table 1), many early lesion interventions raised valid safety, efficacy and ethical concerns. As such, lesions have almost always been considered a treatment of ‘last resort’ and their use has decreased whenever alternative treatments have become available (Fig. 1). For example, the introduction of antipsychotics led to the decline of frontal lobotomy and limbic leucotomy for psychiatric disease while the introduction of l-DOPA led to the decline of lesions for Parkinson’s disease.^54-56^ Similarly, the development of modern DBS, which could reversibly modulate traditional lesion targets with similar therapeutic benefit, led to a major decline in lesion-based interventions.^54^ A prominent randomized trial that directly compared ventral intermediate nucleus of thalamus (VIM)-DBS to thalamotomy for treatment of tremor found that both are similarly effective, but VIM-DBS resulted in fewer side-effects.^17^

Despite the advantages and major benefits of medications and DBS, they are not without their own drawbacks. Antipsychotics can cause extrapyramidal side effects and l-DOPA can cause dyskinesias.^56,57^ DBS is relatively expensive, requires specialized centres, and includes frequent doctor visits, permanent indwelling hardware, battery charging or surgical battery replacements, and risks of intracranial haemorrhage, infection and device malfunctions, which can even lead to severe and sudden clinical deteorioration.^58^ Ethical considerations include possible future financial issues preventing/delaying replacement of the pulse generator and a lifelong reliance on individuals in the companies producing the devices.^59^ Although DBS is currently the dominant technique in functional neurosurgery and the risk/benefit ratio of DBS is expected to continue improving, lesions have remained an option for selected patients and has motivated ongoing research into lesion-based treatments.^60,61^ Use of traditional therapeutic lesioning where DBS is not available or logistically or financially feasible might also allow more patients to benefit from functional neurosurgery.

### Recent advances in therapeutic lesions

**Figure 3:**
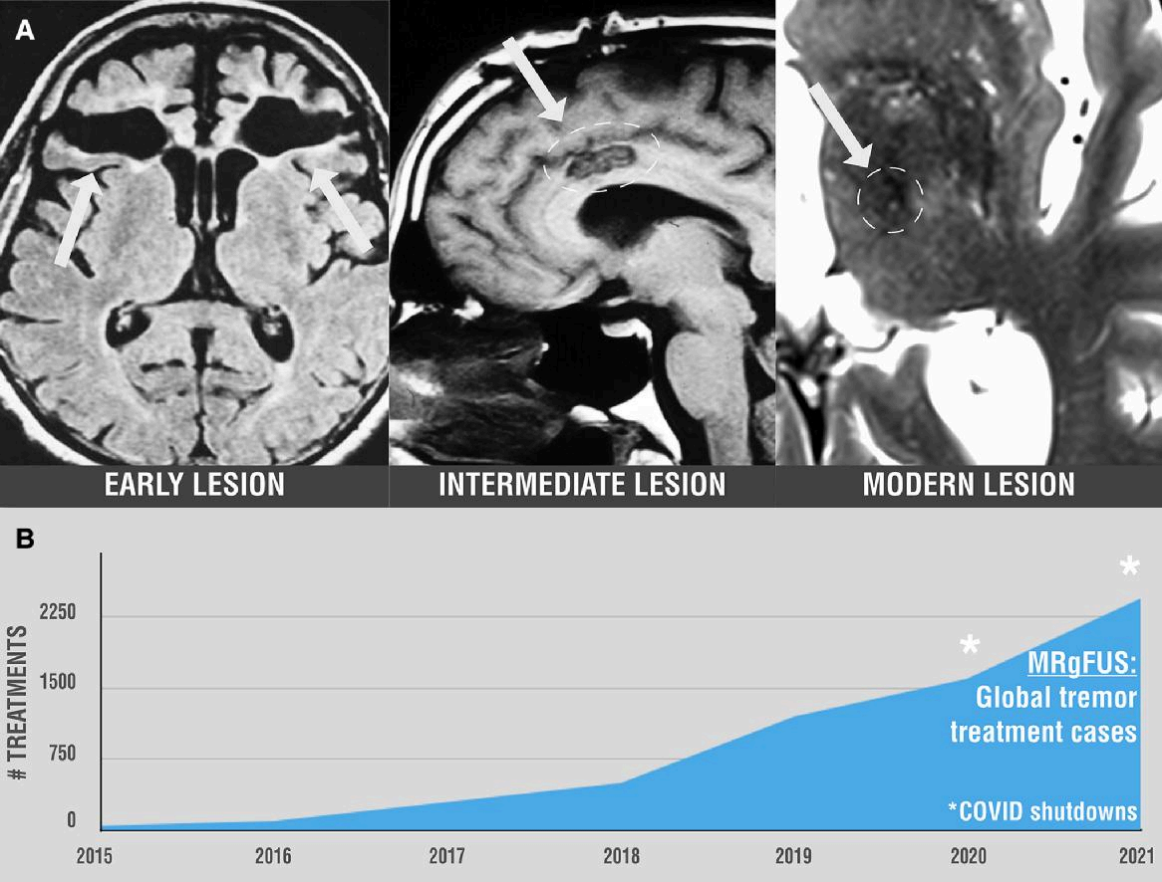
**Evolution of therapeutic lesions in functional neurosurgery**. (**A**) Examples of lesions from different time points in history. Early ablative lesions such those resulting from prefrontal leucotomy (*left*) were large and spanned multiple brain regions.^62^ After introduction of stereotactic frames around 1947, lesions became more precise, such as those generated by stereotactic cingulotomy (*middle*). Current lesions generated by modern ablative surgery are small, precise, and even barely visible on MRI just 3 months after the procedure, such as lesions created by magnetic resonance-guided focused ultrasound (MRgFUS) (*right*). Frontal lobotomy: Image distributed under a CC-BY-3.0 license, courtesy to Frank Gaillard. Cingulotomy: Image distributed under a CC Attribution-Share Alike 4.0 license, courtesy of operativeneurosurgery.com. (**B**) The number of MRgFUS procedures for tremor have increased rapidly over the past few years. Data provided by InSightec for the commercial neuro-exablate system.

Therapeutic lesions have evolved over time to be smaller and more accurate with the goal of improving benefit while reducing off-target side-effects (Fig. 3A). Technical advances have improved our accuracy of reaching lesion targets, including stereotactic frames, real-time MRI and robotic technology.^63^ These latter advances are a major improvement over ventriculography, the targeting technology used in the landmark head-to-head trial of DBS versus lesion therapy.^17^ More refined surgical tools to create lesions such as radiofrequency (RF) ablations, laser ablations and gamma knife can help minimize tissue damage and the invasiveness of lesion-based procedures.^64-66^ A recent advance in lesion treatments is magnetic resonance-guided focused ultrasound (MRgFUS), which may have advantages relative to other lesion-based therapies.^67^ Unlike RF ablations, MRgFUS can be used to create lesions under direct MRI guidance without skin incision or opening the skull.^68,69^ This leads to reduced risk of infection or bleeding, reduced pain, and more rapid surgical recovery.^68,69^ MRgFUS can also be used to create a transient test lesion with immediate clinical effects, an advantage compared to gamma knife in which clinical effects may not appear until months after the treatment.^67^ As such, there has been a consistent increase in the number of MRgFUS operations for each of the past 7 years (Fig. 3B).^68^

### Focused ultrasound: current state

MRgFUS is based on the use of multiple ultrasound beams using a hemispheric distribution of phased arrays combined with real-time MRI-based targeting and thermometry. Lesioning is achieved via ultrasonic energy absorption in brain tissue which in turn reaches critical temperatures that cause neuronal cell death, resulting in controlled thermo-ablation of the target. When using lower energy, MRgFUS is also capable of creating a temporary lesion which can help to probe for an optimal target in a patient, for example, detecting tremor arrest and avoiding side-effects during a thalamotomy.^68,69^ Currently, MRgFUS is clinically used to target VIM for treatment of tremor in essential tremor and Parkinson’s disease as an alternative to DBS.

The efficacy of MRgFUS thermoablation of the VIM to treat essential tremor has been confirmed in a large prospective randomized controlled trial (RCT).^70,71^ The most common adverse effects MRgFUS were contralateral paraesthesia and ataxia, each affecting approximately one-third of the patients, persisting at 12 months follow up in 9% and 14% of patients, respectively. Follow-up data for VIM MRgFUS at 3–5 years is reassuring, without major loss of benefit or the appearance of new side effects.^72,73^ Smaller RCTs have confirmed that MRgFUS can also be used for treatment of parkinsonian tremor (VIM thalamotomy).^74^

There are several ongoing lines of research aiming to expand the clinical indications for MRgFUS. Recent studies have just led to US Food and Drug Administration (FDA) approval of bilateral thalamotomy in essential tremor and suggested benefit of unilateral lesions for motor symptoms in Parkinson’s disease patients who were not eligible for DBS (subthalamotomy).^74-78^ There also are ongoing studies investigating MRgFUS in dystonic tremor (VIM thalamotomy), dystonia (ventro-oral thalamotomy), Parkinson’s dyskinesias (pallidotomy), obsessive-compulsive disorder (anterior capsulotomy), epilepsy (thalamotomy), depression (anterior capsulotomy) and chronic pain (centrolateral thalamotomy) (Supplementary Table 1).^68^

### Focused ultrasound: current unknowns and limitations

To date, there have been no head-to-head trials comparing MRgFUS to RF ablations, gamma knife or DBS.^67,79^ Comparing response rates between different trials targeting VIM, tremor improvement may be less robust and side effects may be more common following MRgFUS versus DBS.^80^ As such, MRgFUS has so far been only offered unilaterally and often for patients who have contraindications for DBS, do not want DBS, or for whom repeated DBS tuning visits or hardware/battery maintenance could prove challenging.^68,69,79^ However, trials of bilateral lesioning using MRgFUS are already being conducted and staged bilateral thalamotomy has just received approval from the US FDA for treatment of essential tremor.^77,78^

While MRgFUS can achieve high accuracy in placing lesions, it has technical restrictions that currently limit its use.^68^ Skull thickness and morphology prevent use of MRgFUS in approximately one fifth of patients with a low skull density ratio because of not reaching high enough temperatures at the target, tissue heating related side-effects and a higher risk of complications.^81,82^ In addition, MRgFUS can currently only be used to lesion structures near the centre of the brain, as efficiency and accuracy diminishes the further one gets from the centre of the brain.^68^ MRgFUS generates an ellipsoid lesion, but there is limited ability to rotate, refine or shape this ellipse to best match the surgical target.^68^ Currently, MRgFUS is still limited in reaching sufficient accuracy to target specific parts of small nuclei, such as the subthalamic nucleus (STN), where sensorimotor or somatotopic representation of the targeted circuits are located.^83^ Similarly, optimal control of the expansion of the thermal lesion is still an ongoing challenge.^68^

MRgFUS is currently available only in specialized centres and expensive compared to RF ablations and gamma knife, which are more available worldwide. These techniques provide an important alternative for MRgFUS, reaching larger populations and allowing more patients to benefit from functional neurosurgery. As such, RF ablation also has regained scientific interest with recent papers showing e.g. clinical benefit with bilateral thalamotomy in essential tremor and unilateral pallidotomy in dystonia.^84,85^

## The future of brain lesions in medicine

**Figure 4:**
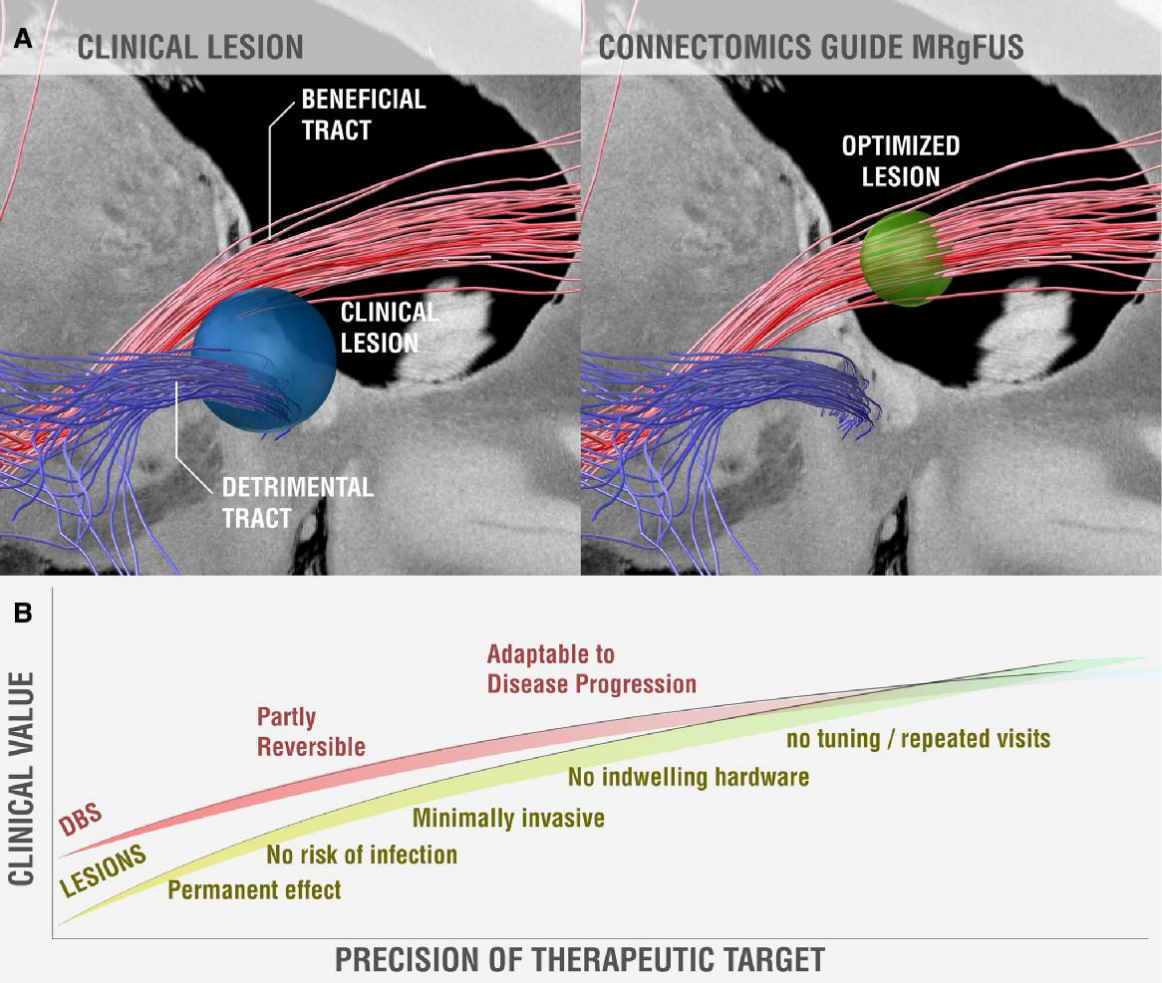
**Schematic illustration of connectomic lesioning**. (**A**) Current clinical lesions (blue sphere) may intersect white matter connections associated with clinical benefit (red fibres) but also detrimental connections associated with less benefit or side effects (blue fibres). In the future, optimized lesions (green sphere) can be guided by connectivity to intersect only beneficial tracts while avoiding the detrimental ones. Red and blue fibre tracts are taken from a recent study of beneficial and detrimental connections for improving obsessive-compulsive disorder (OCD) following deep brain stimulation (DBS) to the anterior limb of the internal capsule.^94^ (**B**) Schematic illustrating how the clinical value of lesions versus DBS could change as the precision of our therapeutic target increases. When the therapeutic target is unclear, DBS has a major advantage over lesions due to reversibility and tunability. As the target and lesioning technique become more precise, this advantage is diminished, and we may reach an inflection point where lesions become preferable over DBS (due to lower infection risk and higher convenience). It should be noted that this schematic does not account for other possible future developments, such as closed-loop DBS, which could increase the benefit of DBS. MRgFUS = magnetic resonance-guided focused ultrasound.

Moving forward, we expect that therapeutic targets will continue to become more precise. First, ongoing efforts to collect large, prospective datasets of spontaneously occurring brain lesions or iatrogenic lesions^86^ should allow us to better map lesion-induced effects to brain regions and brain circuits. The latter will benefit from improved atlases of brain connectivity.^30,87-89^ Second, therapeutic targets may become more precise as we shift from diagnosis-based targets to symptom-specific targets. For example, different targets are already used for different symptoms of Parkinson’s disease such as tremor (VIM), rigidity [STN or globus pallidus interna (GPi)] or dyskinesias (GPi),^90^ while new targets are being investigated for refractory symptoms such as freezing of gait.^91^ Similar symptom-specific targets may prove valuable in other diseases such as depression.^92^ Third, we may be able to reduce side effects as we identify brain circuits to be avoided (Fig. 4A).^93^ For example, one may need to avoid certain connections near the VIM to avoid inducing dysarthria or near the STN to avoid inducing depression, connections which may be distinct from the connections mediating symptom improvement.^16,95^ Finally, therapeutic targets may become more individualized based on individual differences in neuroanatomy. Ongoing advances in neuroimaging (e.g. moving from landmark-based targeting to directly identifying the target structure, facilitated by increasing MRI field strengths, tractography, specialized MRI sequences, etc.) may improve our ability to identify therapeutic targets in individual patients.^96-98^

We also expect that our ability to accurately lesion a target anywhere in the brain will also continue to improve. New advances in MRgFUS design are underway to enlarge the workspace in which lesions can be generated.^68^ Other technological advances may allow for lesioning of specific cell types or specific projections, as well as novel approaches to lesioning in general, such as delivery of cytotoxic agents across the temporarily permeabilized blood–brain barrier.^68^ Finally, ongoing research aims to improve our ability to create transient lesions using hyperthermia, mechanosensitive ion channels, cell membrane capacitance or blood–brain barrier disruption.^68,99^ The ability to create a transient lesion and control the duration of the lesion could allow one to pilot clinical effects prior to a permanent lesion, which would greatly facilitate experimental lesions for new indications.

Given recent advances in lesion mapping, identifying therapeutic targets, and accurately placing lesions at these targets, we are likely to see a continued increase in the use of lesion-based therapies moving forward. These advances may change the benefit-risk ratio of therapeutic lesions relative to competing technologies such as DBS (Fig. 4B). If a precise therapeutic target is known and can be accurately lesioned, traditional advantages of DBS over lesions such as reversibility and tunability could become less important. Conversely, the advantages of lesions such as convenience, lack of indwelling hardware, fewer doctor visits, and reduced infection risk (especially in the case of MRgFUS) may lead to lesions becoming a preferred alternative to DBS in the future. These advantages could prove particularly important in emerging indications such as psychiatric disease, where a lesion may be better tolerated by patients than DBS electrodes, or in geographic areas where DBS and DBS programming are unavailable.

### Cautions for the use of lesions in the future

Although lesions appear to be making a comeback, caution is warranted, especially when using irreversible lesions that could be associated with side effects, or when pursuing psychiatric indications.^79,100^ With permanent lesions, it is important to acknowledge that brain structure and functional organization have interindividual variability and, therefore, a group-level optimal target may not be suitable for all patients.^101^ We also need to learn from the history of lesion-based therapies to avoid repeating the same mistakes.^11^ First, we need to ensure our lesion-based targets are as precise as possible.^100^ Although MRgFUS of the VIM is a US FDA approved therapy for tremor, targeting is usually only based on anatomical landmarks to approximate the location of VIM, and there is debate regarding whether VIM or a different structure near VIM is the ideal target for tremor.^52^ Tunable technologies such as DBS can compensate for some of this uncertainty postoperatively, while lesion-based treatments cannot. Second, we need to ensure that we are minimizing lesion-based side effects. Although acute side effects are often readily apparent, delayed-onset side effects can occur years after surgery. For example, delayed onset ataxia can occur following VIM DBS,^102,103^ and delayed onset cognitive decline can occur following STN DBS.^104^ Both of these side effects can be alleviated with DBS-reprogramming. If these side-effects are associated with the therapeutic target, they might also occur following therapeutic lesions where adjustment is not possible. Finally, informed consent for lesion-based therapies, especially experimental therapies, is critical and should only be conducted under the guidance of independent review panels.

## Summary

Lesion-based localization has sometimes been considered a relic of the past,^8^ but it has re-emerged as a tool for causal localization of symptoms and identification of therapeutic targets. Although there are limitations to lesion-based treatments, new technologies are improving the accuracy, safety, and convenience of therapeutic lesioning. Combined, these developments may facilitate the return of the lesion, complementing the currently available neuroimaging and brain stimulation techniques for localization and therapy.

## Supplementary Material

awad123_Supplementary_Data
