# Supplementary material for: The return of the lesion for localization and therapy
Source: Brain. 2023 Apr 11;146(8):3146–55. doi: 10.1093/brain/awad123 (PMC10393408; doi:10.1093/brain/awad123)
Supplement: awad123_Supplementary_Data [file awad123_supplementary_data.pdf]

***Table S1*. Non-exhaustive list of clinical conditions where lesions have been used therapeutically**

Clinical conditions	Historical targets for lesioning	MRgFUS targets[a]
Parkinson's disease	Cortical motor areas, interior part of the internal capsule, pallidum, fields of Forel, choroidal artery territory, cerebral peduncle, rubrospinal and segmental tracts in the medulla oblongata, spinal cord, lateral cervical spinal cord, STN, thalamus	STN[b], GPi, thalamus (VIM)[b]
Dystonia	GPi, STN, thalamus, subthalamic region, fields of Forel, zona inserta, caudate, dentate nucleus, longitudinal medial fascicle, interstitial nucleus of Cajal, red nucleus, olive nucleus	Thalamus (VIM/Vo)
Essential tremor	Cortical motor areas, internal capsule, cerebral peduncle, caudate, ansa lenticularis, pallidum, zona inserta, posterior subthalamic area, thalamus	Thalamus (VIM)[b], posterior subthalamic area
Epilepsy	Medial temporal lobe (amygdala), thalamus, pallidum, dentate, fornix	Thalamus (ANT)
Pain	Prefrontal cortex (leucotomy), anterior cingulate cortex, mesencephalon (trigemino- or spinothalamic tracts), thalamus, hypothalamus, pituitary, primary sensorimotor cortices	thalamus (CL/VPM/VPL), anterior cingulate cortex
Addiction	Anterior cingulate cortex, Nucleus accumbens, hypothalamus	
OCD	Limbic regions (leucotomy), anterior capsule, anterior cingulate cortex, tracts ventral to the head of the caudate nucleus (subcaudate tractotomy)	anterior limb of the internal capsule
Depression	Limbic regions (leucotomy), anterior capsule, anterior cingulate cortex, tracts ventral to the head of the caudate nucleus (subcaudate tractotomy)	anterior limb of the internal capsule
Eating disorders	Limbic regions (leucotomy), thalamus, nucleus accumbens, anterior limb of the internal capsule	-
Schizophrenia	Frontal lobe, Limbic regions (leucotomy), septal region, tracts ventral to the head of the caudate nucleus (subcaudate tractotomy), anterior cingulate cortex, anterior capsule, hypothalamus, orbitoventromedial undercutting, thalamus	-

For some conditions multiple different targets in the thalamus are listed as "thalamus". Note that the table is not intended as a comprehensive list of all clinical conditions and targets.
ANT = anterior nuclei of thalamus. CL/VPM/VPL = centrolateral/ventroposteromedial/-lateral nucleus of the thalamus.
GPi = globus pallidus interna. OCD = obsessive-compulsive disorder. STN = subthalamic nucleus. VIM = ventrointermediate nucleus of the thalamus. Vo = Ventro-oral nucleus of the thalamus.
[a]Targets lesioned using MR-guided focused ultrasound (MRgFUS).
[b]Efficacy validated in clinical randomized controlled trials.
